# Association between the social isolation and depressive symptoms after the great East Japan earthquake: findings from the baseline survey of the TMM CommCohort study

**DOI:** 10.1186/s12889-021-10896-5

**Published:** 2021-05-15

**Authors:** Yuka Kotozaki, Kozo Tanno, Kiyomi Sakata, Eri Takusari, Kotaro Otsuka, Hiroaki Tomita, Ryohei Sasaki, Nobuyuki Takanashi, Takahiro Mikami, Atsushi Hozawa, Naoki Nakaya, Naho Tsuchiya, Tomohiro Nakamura, Akira Narita, Yasuyuki Taki, Atsushi Shimizu, Jiro Hitomi, Mamoru Satoh, Makoto Sasaki

**Affiliations:** 1 Iwate Tohoku Medical Megabank Organization, Iwate Medical University, 1-1-1 Idaidori, Yahaba, Shiwa, Iwate, 028-3694 Japan; 2 Department of Hygiene and Preventive Medicine, School of Medicine, Iwate Medical University, Iwate, Japan; 3 Department of Neuropsychiatry, School of Medicine, Iwate Medical University, Iwate, Japan; 4 Department of Psychiatry, Graduate School of Medicine, Tohoku University, Sendai, Japan; 5 Tohoku Medical Megabank Organization, Tohoku University, Sendai, Japan; 6 Division of Disaster Medical Science, International Research Institute of Disaster Science, Tohoku University, Sendai, Japan; 7 Division of Physical Education, Department of Human Sciences, Iwate Medical University Center for Liberal Arts and Sciences, Iwate, Japan; 8 Department of Anatomy, School of Medicine, Iwate Medical University, Iwate, Japan; 9 Department of Health Science, Saitama Prefectural University, Koshigaya, Japan; 10 Department of Radiology and Nuclear Medicine, Institute of Development, Aging and Cancer, Tohoku University, Sendai, Japan; 11 Division of Biomedical Information Analysis, Institute for Biomedical Sciences, Iwate Medical University, Iwate, Japan; 12 Division of Ultrahigh Field MRI, Institute for Biomedical Sciences, Iwate Medical University, Iwate, Japan

**Keywords:** Tohoku medical megabank project, Cross-sectional study, Great East Japan earthquake, House damage, Death of family members, Social isolation, Depressive symptoms

## Abstract

**Background:**

Social isolation and mental health issues have become a severe problem in disaster areas in the Great East Japan Earthquake. This study examined whether the combination of the house damage and social isolation or the combination of the death of family members and social isolation is associated with depressive symptoms among survivors using the baseline study data of the Tohoku Medical Megabank Project Community-Based Cohort Study (TMM CommCohort Study).

**Methods:**

We used cross-sectional data from a baseline survey of 48,958 participants (18,423 males, 30,535 females; aged 60.1 ± 11.2 years) to examine the association between social isolation measured by the Lubben social network scale 6 (LSNS-6) and depressive symptoms measured by the Center for Epidemiological Studies-Depressive Scale (CES-D). The presence of social isolation and depressive symptoms was defined by an LSNS-6 score of < 12 and a CES-D score of ≥16, respectively. We performed a logistic regression analysis to determine the multivariable-adjusted odds ratio (95% confidence interval) [AOR (95% CI)] for depressive symptoms according to sex in the social isolation in comparison to without social isolation, and the associations of the combination of the house damage or the death of family members and social isolation and depressive symptoms.

**Results:**

Social isolation was significantly associated with depressive symptoms (males: OR = 1.87; 95% CI = 1.72–2.04, females: OR = 2.13; 95% CI = 2.00–2.26). Both males and females respondents with severe house damage and social isolation had a greater risk of depressive symptoms in comparison to those with an undamaged house and without social isolation (males: OR = 3.40; 95% CI = 2.73–4.24, females: OR = 2.92; 95% CI = 2.46–3.46). The risk of depressive symptoms was also higher in both males and females respondents with the death of family members and social isolation in comparison to those without the death of family members and without social isolation (males: OR = 2.18; 95% CI = 1.90–2.50, females: OR = 2.60; 95% CI = 2.35–2.88).

**Conclusion:**

The findings suggested that a combination of social isolation and severe house damage and the death of family members caused by a large-scale natural disaster was associated with a higher risk of depressive symptoms although the interaction was not statistically significant.

**Supplementary Information:**

The online version contains supplementary material available at 10.1186/s12889-021-10896-5.

## Introduction

The Great East Japan Earthquake (GEJE) erupted in the northeastern region of Japan at 14:46 JST on Friday, March 11, 2011. This 9.0-magnitude earthquake was followed by enormous tsunamis and caused significant damage, primarily on the northeastern Pacific coast of Japan in locations such as Iwate, Miyagi, and Fukushima. At 1 month after the earthquake, more than 10,000 people had died, and more than 10,000 people were missing [1, 2]. At 10 years after the earthquake, as of March 11, 2021, 2525 people were still missing [3]. Many survivors lost family and friends to the earthquake and tsunamis and also were living in shelters after their houses were damaged, putting them at risk of social isolation.

Social isolation is an objective and quantifiable outcome of reduced social network size [4, 5] {Cacioppo, 2003 #2;Steptoe, 2013 #3}. Social networks represent the structural aspects of social relationships and objective characteristics such as size, frequency, and density [4]. In social networks, the quality of friendships and family relationships is known to be important [6]. In addition, postdisaster social isolation precipitates the immediate and delayed impact of disaster stress [7]. Furthermore, severely traumatized survivors experience social isolation [8]. Epidemiological reports on the Japanese population have shown that the percentage of social isolation increased after the GEJE [9, 10]. Studies have also shown that socially isolated individuals are at an increased risk for the development of cardiovascular diseases [11, 12], infectious diseases [13, 14], cognitive decline [15, 16], and depressive symptoms [17, 18].

Depressive symptoms are common and serious in illness and negatively impact emotions, thinking, and behavior. These symptoms can lead to many emotional and physical problems and decrease the ability to function at work and home [19]. Depressive symptoms affect an estimated 1 in 15 adults (6.7%) in any given year, and 1 in 6 adults (16.6%) will experience depression at some time in life [20]. Depression is also approximately twice as likely to occur in females than in males [20, 21]. Studies have shown that depressive symptoms increased after the earthquake [9, 22, 23]. It has also been reported that house damage and the death of family members were associated with depressive symptoms after the earthquake [24–26]. However, few reports have evaluated the association of social isolation and depressive symptoms due to the severity of house damage and the death of family members.

Therefore, this study aimed to investigate whether social isolation is associated with depressive symptoms and whether the combination of house damage and social isolation or the combination of the death of family members and social isolation is associated with depressive symptoms among community residents living in areas affected by the GEJE.

## Methods

### Study population

This study is a part of the Tohoku Medical Megabank Project Community-Based Cohort Study (TMM CommCohort Study). Our research team has conducted the TMM CommCohort Study since 2013 to investigate the health of residents affected by conditions after the GEJE [27, 28]. The TMM CommCohort Study investigates various mental health and lifestyle habits, including those caused by damage due to the earthquake, in residents of the Iwate and Miyagi prefectures. In the TMM CommCohort Study, we recruited participants through two approaches. The type 1 survey data were collected at specific health check-up sites. The type 2 survey data were obtained at community support centers or satellite sites [28]. We used the type 1 survey data to conduct our analysis.

**Fig. 1:**
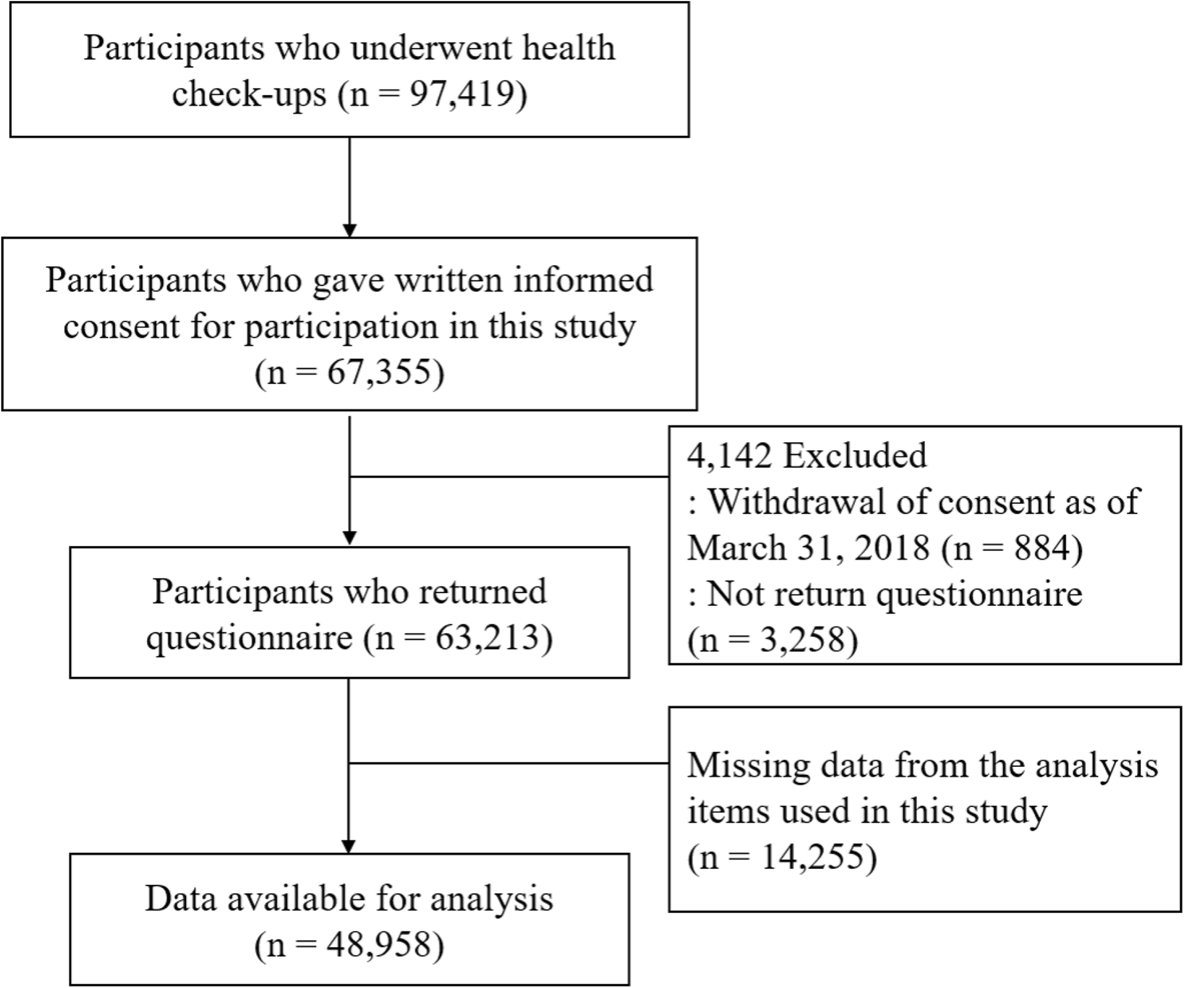
Flow diagram of the participants

The data on study participants are shown in Fig. 1. A total of 97,419 participants underwent health checkups, and 67,355 participants gave written informed consent for participation in the study according to Declaration of Helsinki (1991) guidelines (consent rate, 70.0%). Among these, we excluded 3258 participants who did not return the questionnaire, 884 participants who withdrew consent as of March 31, 2018 [28], and 13,126 participants with missing data on items analyzed in this study. Ultimately, the dataset included 48,958 participants (18,423 males and 30,535 females, aged 60.1 ± 11.2 years). The Ethics Committee of Tohoku Medical Megabank Organization (ToMMo) (first approval, 2012–4-617; most recent approval, 2018–4-087) and Iwate Medical University (HGH25–2) approved all study procedures.

### Measurements

#### Depressive symptoms

Depressive symptoms were assessed using the Center for Epidemiological Studies–Depressive Scale (CES-D) [29]. This scale consists of 20 items including 16 negative items that indicate the presence of symptoms (7 psychological symptoms items, 7 physical symptoms items, and 2 interpersonal relations items) and 4 positive items that indicate the absence of symptoms. The study also used a 4-point Likert scale. The reliability and validity of the Japanese version of the CES-D have been confirmed [30]. It shows high sensitivity (100%) and specificity (69.4%) and a positive predictive value (30.6%) in random samples of community residents [31]. We determined the presence of depressive symptoms by a CES-D score of ≥16 [29, 30].

#### Social isolation

Social isolation was assessed using the Lubben Social Network Scale-6 (LSNS-6) [4, 32]. The LSNS-6 consists of 6 items on social connections (3 questions about family ties and 3 questions about friendship ties). Each item is rated on a 6-point Likert scale. The LSNS-6 ranges from 0 to 30. The reliability and validity of the Japanese version of the LSNS-6 have been confirmed [33]. Social isolation is defined by a score of < 12 [32, 33].

#### The severity of house damage and death of family members due to the GEJE

We used 6 options to assess house damage caused by the GEJE: (1) totally damaged (including all outflows); (2) seriously damaged; (3) half-damaged; (4) partially damaged; (5) no damage; and (6) nonresidence. We further classified these options as totally damaged, half-damaged (seriously damaged, half-damaged, and partially damaged), or undamaged (no damage or nonresidence). It is very likely that those with house damage were evacuated to temporary housing, which may have excluded them from their community. Even if some communities moved to temporary accommodating areas altogether, maintaining social contact could be reduced.

Participants responded *yes* or *no* to inquiries about the death of family members due to the GEJE.

#### Covariates

The following demographic characteristics were included in the analysis as covariates: age (continuous); survey year (2013, 2014, or 2015); and inland or coastal area, with inland area indicating that the participant’s municipality was not shown on a map as bordering a sea and coastal area indicating that the participant’s municipality was shown on a map as bordering a sea. The following factors were also included as covariates in the analysis because studies have suggested that these factors are associated with depression and social isolation [5, 34–37]: education level (junior high school, high school, college, university, or higher); marital status (unmarried or married); number of household members (living alone or ≥ 2); work status (unemployed or employed); smoking habits (nonsmoker or current smoker); drinking habits (nondrinker or current drinker); past or current major illness (hypertension, diabetes mellitus, hyperlipidemia, cancer, coronary artery disease, or stroke); body mass index (< 18.5, 18.5 to < 25, or ≥ 25 kg/m^2^); and insomnia. Insomnia was determined by a score of ≥6 on the Athens Insomnia Scale [38, 39].

### Statistical analysis

We analyzed the data from both sexes separately because it has been reported that females experience more depressive symptoms than males [20, 21]. Continuous variables were summarized as the mean and standard deviation, and categorical variables were summarized as percentages. A chi-squared test (for linear trend) for categorical variables and student’s t-test for continuous variables were used to evaluate differences in characteristics. We also compared sociodemographics between participants and nonparticipants, the proportion of depressive symptoms by survey year and area between the Iwate and Miyagi prefectures, and the proportion of depressive symptoms by month and season.

We used a multivariable logistic regression analysis to analyze the association between depressive symptoms and social isolation. We also applied stratified analyses. We calculated the OR and 95% CI for depressive symptoms according to social isolation by survey year and age group (< 65 or ≥ 65 years of age). The model used for these analyses was the same as the models calculated earlier. In addition, to investigate the impact of the combination of damage due to the GEJE and social isolation on depressive symptoms, we divided the participants into six groups according to the combination of the severity of house damage and social isolation and determined the ORs and 95% CIs for depressive symptoms in five groups compared with the group without house damage or social isolation. Similarly, participants were classified into four groups based on the combination of death of family members caused by the earthquake and social isolation and determined the ORs and 95% CIs for depressive symptoms in three groups compared with the group without the death of family members or social isolation. Interaction terms were also considered in the stratified analysis.

Moreover, because the percentage of missing data among those who returned the questionnaire (*n* = 63,213) was 22.6% (*n* = 14,255), we used Markov chain Monte Carlo methods based on a linear regression model and corrected for predictive mean matching to impute item-level missing data. The results across five imputed datasets were combined by averaging.

All statistical analyses were conducted using SPSS version 25.0 for Windows (IBM, Tokyo, Japan). *P* values of < 0.05 indicated statistical significance.

## Results

In the overall study population, the prevalence of social isolation was 28.8% in males and 23.9% in females, and the prevalence of depressive symptoms was 19.8% in males and 28.7% in females. The characteristics of the participants according to the presence or absence of social isolation are shown in Table 1. In both sexes, the proportion of depressive symptoms was significantly higher in participants who had social isolation than in those without social isolation; 30.0% vs. 15.7%, respectively, in males and 43.6% vs. 24.0% in females. The proportions of participants whose houses were totally damaged and with the death of one or more family member due to the GEJE were likely to be higher among participants who had social isolation than among those without social isolation in both sexes. In comparison to participants without social isolation, in both sexes, participants with social isolation were also younger, more likely to live in a coastal area, be unmarried, live alone, be a current smoker, have a low BMI, and have insomnia. Furthermore, only females were likely to difference in proportions of unemployed status.

**Table 1:** Characteristics of the participants according to the presence or absence of social isolation by sex (*n* = 48,958)

		Males (*n* = 18,423)			Females (*n* = 30,535)		
		Nonsocial Isolation (*n* = 13,114)	Social Isolation (*n* = 5309)	*P* Value	Nonsocial Isolation (*n* = 23,240)	Social Isolation (*n* = 7295)	*P* Value
Age (continuous)		62.7 (10.3)	60.7 (10.4)	< 0.001*	59.4 (11.4)	57.1 (11.2)	< 0.001*
Survey year (%)	2013	23.7	21.5	0.007	24.1	22.2	0.003
	2014	41.4	42.5		41.4	42.6	
	2015	34.9	36.0		34.5	35.3	
Area (%)	Inland	48.4	46.6	0.031	45.3	44.1	0.072
	Coast	51.6	53.4		54.7	55.9	
Depressive symptoms (%)		15.7	30.0	< 0.001	24.0	43.6	< 0.001
Severity of house damage (%)	Undamaged	43.9	47.5	< 0.001**	45.0	50.3	< 0.001**
	Half-damaged	46.5	42.7		45.0	39.4	
	Totally damaged	9.6	9.8		10.0	10.4	
Death of family members due to the GEJE (%)	One or more	38.1	42.7	< 0.001	36.2	42.0	< 0.001
Education level (%)	Junior high school	22.4	22.0	0.039	17.2	18.0	0.141
	High school	50.8	49.3		48.7	49.1	
	College, university, and higher	26.0	28.0		33.4	32.3	
	Other	0.8	0.7		0.7	0.6	
Marital status (%)	Unmarried	14.5	28.8	< 0.001	19.9	25.5	< 0.001
Number of household members (%)	living alone	5.3	11.4	< 0.001	7.3	8.4	0.002
Working status (%)	Unemployed	59.5	54.2	< 0.001	43.9	46.9	< 0.001
Smoking habits (%)	Smoker	26.5	25.4	0.137	5.8	8.1	< 0.001
Drinking habits (%)	Drinker	76.5	70.0	< 0.001	35.6	34.3	0.034
Past or current major illness (%)	Hypertension	33.8	33.4	0.62	22.9	19.8	< 0.001
	Diabetes mellitus	8.0	7.9	0.769	3.3	3.6	0.201
	Hyperlipidemia	9.7	10.9	0.011	13.7	12.5	0.009
	Cancer	8.6	7.5	0.009	6.8	6.9	0.951
	Coronary artery disease	4.8	5.2	0.201	1.6	1.2	0.023
	Stroke	3.7	3.8	0.708	1.7	1.6	0.449
BMI (%)	< 18.5 kg/m^2^	2.0	2.7	< 0.001	7.6	10.1	< 0.001
	18.5 to < 25.0 kg/m^2^	62.1	64.0		67.2	66.3	
	≥25.0 kg/m^2^	35.8	33.3		25.2	23.6	
Insomnia (%)		13.7	24.6	< 0.001	22.5	34.9	< 0.001

Depressive symptoms, CES-D ≥ 16; social isolation, LSNS-6 < 12; insomnia, AIS ≥ 6

*CES-D* Center for Epidemiologic Studies Depression Scale, *LSNS-6* Lubben Social Network Scale-6, *AIS* Athene Insomnia Scale, *GEJE* Great East Japan Earthquake, *BMI* body mass index

*P* value: tested by chi-square test; * tested by student t-test; ** tested by chi-square test for linear trend

Statistical significance, *P* < 0.05

The comparison of sociodemographics between participants and nonparticipants is shown in Supplemental Table 1. There were no significant differences between participants and nonparticipants except for past or present hypertension.

The proportion of depressive symptoms by survey year and area between the Iwate and Miyagi prefectures is shown in Supplemental Table 2. The differences in the proportions were observed by survey year and area. The combination of survey year and area was used as a dummy variable to be adjusted in subsequent analyses.

The proportion of depressive symptoms by month and season is shown in Supplemental Table 3a and b. The highest percentage of depressive symptoms was 26.3% in May and December, and the lowest percentage was 20.5% in January. However, there was no significant difference in the proportion of depressive symptoms by season (*P* = 0.740).

The adjusted ORs (AOR [95% CIs]) for depressive symptoms according to house damage, death of family members, and social isolation are shown in Table 2. House damage was significantly associated with depressive symptoms in both sexes after adjusting for all covariates (1.69 [1.46–1.94] in males and 1.24 [1.16–1.32] in females). The death of family members and social isolation were significantly associated with depressive symptoms in both sexes after adjusting for all covariates (death of family members, 1.16 [1.06–1.27] in males and 1.24 [1.16–1.32] in females; social isolation, 1.87 [1.72–2.04] in males and 2.13 [2.00–2.26] in females).

**Table 2:** Adjusted ORs (95% CI) of depressive symptoms according to house damage, death of family members due to the GEJE, and social isolation by sex

	Male (*n* = 18,423)				Female (*n* = 30,535)			
	No. of Cases with Depressive Symptoms / No. of subjects	OR	95% CI	*P* Value	No. of cases with Depressive Symptoms / No. of Subjects	OR	95% CI	*P* Value
House damage								
Undamaged	1487 / 8286	1.00	reference		3697 / 14,128	1.00	reference	
Half-damaged	1636 / 8363	1.20	1.10–1.32	< 0.001	3880 / 13,320	1.18	1.11–1.25	< 0.001
Totally damaged	523 / 1774	1.69	1.46–1.94	< 0.001	1174 / 3087	1.48	1.34–1.63	< 0.001
Death of family members due to the GEJE	1557 / 7188	1.16	1.06–1.27	0.002	5074 / 18,964	1.24	1.16–1.32	< 0.001
Social isolation	1592 / 5309	1.87	1.72–2.04	< 0.001	3177 / 7295	2.13	2.00–2.26	< 0.001

*OR* odds ratio, *95% CI* 95% confidence interval

Depressive symptoms, CES-D ≥ 16; social isolation, LSNS-6 < 12

Adjusted for age; survey year; area; education level; marital status; number of household members; working status; smoking habits; drinking habits; past or current major illness; BMI; AIS; and survey year*area

Statistical significance, *P* < 0.05

The AORs (95% CI) for depressive symptoms according to social isolation, stratified by survey year, are shown in Supplemental Table 4. There was no interaction between survey year and social isolation in either males or females. The AORs (95% CI) of depressive symptoms according to social isolation, stratified by age group, are shown in Supplemental Table 5. There was an interaction between age group and social isolation in males (*P* = 0.005). Conversely, there was no interaction between age group and social isolation in females.

The AORs (95% CIs) for depressive symptoms according to the severity of house damage and social isolation are shown in Table 3. In both males and females, the risk of depressive symptoms increased depending on the severity of house damage or social isolation compared with the group with no house damage or social isolation. The OR was highest for those with total house damage and social isolation (OR [95% CI], 3.40 [2.73–4.24] in males and 2.92 [2.46–3.46] in females). There was no interaction between the severity of house damage and social isolation in either males or females (*P* = 0.402 in males and *P* = 0.451 in females). The AORs (95% CI) for depressive symptoms according to the presence or absence of death of family members caused by the GEJE and social isolation are shown in Table 4. Both males and females showed an increased risk of depressive symptoms according to the presence of death of family members or social isolation due to the GEJE. The OR was highest for the death of family members due to the GEJE and social isolation (OR [95% CI], 2.18 [1.90–2.50] in males and 2.60 [2.35–2.88] in females). There was no significant interaction between the death of family members due to the GEJE and social isolation in either males or females (*P* = 0.886 in males and *P* = 0.612 in females).

**Table 3:** Adjusted ORs (95% CI) of depressive symptoms according to the severity of house damage and social isolation

Severity of house damage × Social isolation	Males (*n* = 18,423)					Females (*n* = 30,535)				
	No. of Cases with Depressive Symptoms / No. of Subjects	OR	95% CI	*P* Value	*P* for Interaction	No. of Cases with Depressive Symptoms / No. of Subjects	OR	95% CI	*P* Value	*P* for Interaction
Undamaged × Nonsocial isolation	853 / 5833	1.00	reference		0.402	2343 / 10,680	1.00	reference		0.451
Half-damaged × Nonsocial isolation	974 / 6191	1.18	1.06–1.32	0.004		2658 / 10,658	1.17	1.08–1.25	< 0.001	
Totally damaged ×Nonsocial isolation	301 / 1284	1.58	1.33–1.87	< 0.001		796 / 2370	1.51	1.35–1.69	< 0.001	
Undamaged × Social isolation	723 / 2612	1.79	1.57–2.03	< 0.001		1562 / 3812	2.10	1.92–2.30	< 0.001	
Half-damaged × Social isolation	746 / 2357	2.23	1.95–2.54	< 0.001		1398 / 2985	2.57	2.33–2.83	< 0.001	
Totally damaged × Social isolation	241 / 523	3.40	2.73–4.24	< 0.001		417 / 782	2.92	2.46–3.46	< 0.001	

*OR* odds ratio, *95% CI* 95% confidence interval

Depressive symptoms, CES-D ≥ 16; social isolation, LSNS-6 < 12

Adjusted for age; survey year; area; education level; marital status; number of household members; working status; smoking habits; drinking habits; past or current major illness; BMI; AIS; death of family members due to the GEJE; and survey year*area

Statistical significance, *P* < 0.05

**Table 4:** Adjusted ORs (95% CI) of depressive symptoms according to the death of family members due to the GEJE and social isolation

Death of family members due to the GEJE╳Social isolation	Males (*n* = 18,423)					Females (*n* = 30,535)				
	No. of Cases with Depressive Symptoms / No. of Subjects	OR	95% CI	*P* Value	*P* for Interaction	No. of Cases with Depressive Symptoms / No. of Subjects	OR	95% CI	*P* Value	*P* for interaction
No death of family members due to the GEJE ╳nonsocial isolation	1088 / 7691	1.00	reference		0.886	3060 / 14,088	1.00	reference		0.612
Death of family members due to the GEJE ╳nonsocial isolation	966 / 5423	1.16	1.03–1.29	0.01		2514 / 9152	1.25	1.16–1.34	< 0.001	
No death of family members due to the GEJE ╳social isolation	1001 / 3544	1.86	1.67–2.08	< 0.001		2014 / 4876	2.15	1.99–2.32	< 0.001	
Death of family members due to the GEJE ╳social isolation	591 / 1765	2.18	1.90–2.50	< 0.001		1163 / 2419	2.60	2.35–2.88	< 0.001	

*OR* odds ratio, *95% CI* 95% confidence interval

Depressive symptoms, CES-D ≥ 16; social isolation, LSNS-6 < 12

Adjusted for age; survey year; area; education level; marital status; number of household members; working status; smoking habits; drinking habits; past or current major illness; BMI; AIS; severity of house damage; and survey year*area

Statistical significance, *P* < 0.05

The AORs (95% CI) for depressive symptoms according to the severity of house damage and social isolation based on the multiply imputed datasets are shown in Supplemental Table 6. House damage was significantly associated with depressive symptoms in both sexes after adjusting for all covariates (1.61 [1.53–1.70] in males and 1.43 [1.12–1.49] in females). The death of family members and social isolation was significantly associated with depressive symptoms in both sexes after adjusting for all covariates (death of family members, 1.15 [1.11–1.19] in males and 1.24 [1.21–1.27] in females; social isolation, 1.90 [1.84–1.96] in males and 2.14 [2.09–2.20] in females).

The AORs (95% CI) for depressive symptoms according to social isolation, stratified by survey year, are shown in Supplemental Table 7. There was no interaction between survey year and social isolation in either males or females. The AORs (95% CI) of depressive symptoms according to social isolation, stratified by age group, are shown in Supplemental Table 8. There was an interaction between age group and social isolation in males (*P* = 0.005). Conversely, there was no interaction between age group and social isolation in females.

The AORs (95% CI) for depressive symptoms according to the severity of house damage and social isolation based on the multiply imputed datasets are shown in Supplemental Table 9. In both males and females, the risk of depressive symptoms increased depending on the severity of house damage or social isolation compared with the group with no house damage or social isolation. The OR was highest for those with total house damage and social isolation (OR [95% CI] 3.20 [2.94–3.48] in males and 2.87 [2.68–3.07] in females). There was no interaction between the severity of house damage and social isolation in either males or females (*P* = 0.442 in males and *P* = 0.407in females). The AORs (95% CI) for depressive symptoms according to the presence or absence of the death of family members due to the GEJE and social isolation based on the multiply imputed datasets are shown in Supplemental Table 10. Both males and females showed an increased risk of depressive symptoms according to the death of family members or social isolation due to the earthquake. The OR was highest for the death of family members due to the GEJE and social isolation (OR [95% CI] 2.20 [2.09–2.32] in males and 2.67 [2.56–2.78] in females). There was no significant interaction between the death of family members due to the GEJE and social isolation in either males or females (*P* = 0.853 in males and *P* = 0.543 in females). A comparison of the results of the multiple imputation with those of the complete case showed no significant difference.

## Discussion

We showed that socially isolated individuals had approximately twice the risk of experiencing depressive symptoms compared with those who were not socially isolated at 3 to 5 years after the GEJE in both males and females. In addition, the combination of house damage and social isolation due to the GEJE and the combination of family death and social isolation due to the GEJE were significantly associated with depressive symptoms in males and females.

Our data show that the prevalence of social isolation at 3 to 5 years after the GEJE was 28.8% in males and 23.9% in females. The LSNS-6 was commonly used to assess social isolation after the GEJE. Yokoyama et al. reported that 41.6% of residents living in the heavily affected Iwate prefecture experienced social isolation at 6 months to 1 year after the earthquake [9]. In a survey of victims in the coastal areas of the Miyagi prefecture, Sone et al. also reported that 24.9 and 26.0% of survivors were socially isolated at 1 and 3 years, respectively, after the earthquake [10]. Although the assessment timing varied in previous studies, the prevalence of social isolation after the earthquake was approximately 30%.

In our study, the prevalence of depressive symptoms as assessed by the CES-D was 19.8% in males and 28.7% in females at 3 to 5 years after the GEJE. Because CES-D depressive symptoms in Japan are estimated to occur in one in 15 adults (6.7%) in a single year and that one in six adults (16.6%) will experience depression at some point in life [20], the prevalence of depressive symptoms in this study is high. Yokoyama et al. used the Kessler 6 to assess mental health at 6 months to 1 year after the earthquake and reported that the proportion of individuals with poor mental health or depressive symptoms was 42.6% [9]. Matsubara et al. used the Patient Health Questionnaire-2 to assess depressive symptoms at 2 to 4 months after the earthquake and reported that the prevalence was 8.1% [23]. Tsuboya et al. assessed depressive symptoms before and after the earthquake using the Geriatric Depression Scale and reported a significant increase of 1.22 points in depressive symptom scores 3 years after the earthquake compared with before the earthquake [25]. However, there are no known reports of depressive symptoms after the earthquake using specific measurement tools such as the CES-D that comprehensively assess the main depressive symptoms, including psychological symptoms, physical symptoms, interpersonal relationships, and positive mood. Incidentally, the CES-D was used to examine depressive symptoms in the 1993 Midwest Floods [40] and the 2008 torrential rains in the mideastern region of the Korean peninsula [41]. The prevalence of depressive symptoms in these studies was 9.5 and 45.4%, respectively [40, 41]. Although our results show an intermediate prevalence compared with these studies, we think that the data are important for the accumulation of knowledge.

Regarding the association between social isolation and depressive symptoms, a previous study showed that predisaster social support can prevent the onset of postdisaster depression [42]. In this study, Sasaki et al. found that more social support before the disaster reduced the risk of developing depressive symptoms after the disaster. Conversely, our study shows that postdisaster social isolation is associated with depressive symptoms and that a combination of social isolation and severe house damage and the death of family members caused by a large-scale natural disaster may be associated with a higher risk of depressive symptoms. The points noted above show the differences between the two studies.

Studies have reported that social isolation and depressive symptoms after a disaster were linked to house damage and the death of family members [6, 24–26, 43, 44]. Property damage caused by a natural disaster and changes to the living environment, such as temporary housing after a disaster, severed social connections and contributed to social isolation, which affected the mental health of survivors [43]. A national, longitudinal survey conducted after the 1999 Chi-Chi earthquake reported that people whose houses were damaged during the disaster were at risk of experiencing depressive symptoms and that socially isolated individuals experienced more depressive symptoms [44]. Social isolation can also be caused by the death of family members [6]. Other studies have reported an association between depressive symptoms and severe house damage [24–26] and the death of family members [26]. Some whose houses were severely damaged or who lost family members in the Great East Japan Earthquake had to cut social ties developed in the neighborhoods where they lived and make new ties. However, individuals in this situation may have become increasingly isolated due to a sense of entrapment, difficulty in interacting with others, or a decrease in their attempts to connect with others due to the earthquake. Continued social isolation may make it increasingly difficult to communicate with others, and an increase in unresolved anxiety and worry in daily life may lead to mental instability and depressive symptoms. Our results are important in reporting depressive symptoms in people with social isolation caused by house damage or the death of family members after a large-scale disaster.

Although various organizations have provided extensive mental health and psychosocial support after catastrophic events such as the Great East Japan Earthquake, the lack of strategies for such support has been suggested to be a major problem [45]. People who cannot make housing plans after a catastrophic event are at high risk of psychological distress in the year after the disaster, suggesting the need for their psychological support [46]. However, it is difficult for local governments to intervene in the community. This study may provide evidence to suggest that psychological and social support needs to be provided as early as possible for people who have experienced house damage or the death of family members to help avoid the development of mental health problems. Local governments need to provide strategic psychological support for people who have experienced house damage or the death of family members in collaboration with relevant organizations, medical professionals, and the community.

This study had several limitations. First, as a cross-sectional study, it was unable to show causal relationships among depressive symptoms. Second, the lack of predisaster data made it impossible to determine whether the participants had social isolation before the GEJE. Third, the independent effects of social isolation, the death of family members, and house damage cannot be determined in this study design because these effects were consequences of the earthquake. Fourth, participants analyzed in this study had participated in the TMM CommCohort Study. This target population may have had greater health awareness and a better health status compared with the general population in the target area, and thus, the prevalence of social isolation and odds ratios for depressive symptoms may have been underestimated. Fifth, the damage caused by the earthquake was self-reported, and the responses may have been inaccurate. It is difficult to determine the actual damage caused by the earthquake to the participants at this stage. Finally, because the study area was limited, the generalization of results must be considered carefully. However, this study is significant because few known studies have reported an association between social isolation and depressive symptoms after an earthquake using a population-based cohort study design and a large sample.

## Conclusion

Survivors of the Great East Japan Earthquake with social isolation were more likely to have significant depressive symptoms compared with those without social isolation. We also found that the risk of depressive symptoms among socially isolated people was increased by the severity of house damage and the death of family members due to the earthquake. The impact of disasters on the mental health of people who are susceptible to social isolation warrants attention. People who are prone to social isolation, especially those who have experienced severe house damage or the death of family members, may require both medium- and long-term care and psychosocial support.

## Supplementary Information

**Additional file 1: Supplemental Table 1.** Comparison of sociodemographics between participants and nonparticipants. **Supplemental Table 2.** Proportion of depressive symptoms by survey year and area between Iwate and Miyagi prefectures. **Supplemental Table 3.** Proportion of depressive symptoms monthly and by season. **Supplemental Table 4.** Adjusted ORs (95% CI) of depressive symptoms according to social isolation by survey year. **Supplemental Table 5.** Adjusted ORs (95% CI) of depressive symptoms according to social isolation by age group. **Supplemental Table 6.** Adjusted ORs (95% CI) of depressive symptoms according to house damage, death of family members, and social isolation by sex by analyzing multiply imputed datasets. **Supplemental Table 7.** Adjusted ORs (95% CI) of depressive symptoms according to social isolation by survey year by analyzing multiply imputed datasets. **Supplemental Table 8.** Adjusted ORs (95% CI) of depressive symptoms according to social isolation by age group by analyzing multiply imputed datasets. **Supplemental Table 9.** Adjusted ORs (95% CI) of depressive symptoms according to the severity of house damage and social isolation by analyzing multiply imputed datasets. **Supplemental Table 10.** Adjusted ORs (95% CI) of depressive symptoms according to death of family members due to the GEJE and social isolation by analyzing multiply imputed datasets.

## Data Availability

The TMM cohort data is available upon approval by Sample and Data Access Committee of Tohoku Medical Megabank Project. For more information, visit http://www.dist.megabank.tohoku.ac.jp/.

## Abbreviations

AIS: Athens Insomnia Scale
BMI: Body Mass Index
CES-D: The Center for Epidemiologic Studies Depression Scale
CI: Confidence interval
GEJE: Great East Japan Earthquake
LSNS-6: Lubben Social Network Scale 6
OR: Odds ratio
SD: Standard deviation
SPSS: Statistical package for social science
ToMMo: Tohoku Medical Megabank Organization

## References

[1] Reconstruction Design Council in response to the Great East Japan Earthquake. Towards Reconstruction: Hope beyond the Disaster: report to the Prime Minister of the Reconstruction Design Council in response to the Great East Japan Earthquake. 2011. Available at: https://warp.da.ndl.go.jp/info:ndljp/pid/3508860/www.reconstruction.go.jp/topics/%E6%8F%90%E8%A8%80%EF%BC%88%E8%8B%B1%E8%AA%9E%EF%BC%89.pdf.

[2] Furukawa K, Arai H (2011). Earthquake in Japan. Lancet.

[3] National Police Agency (2021). Police measures and damage caused by the Great East Japan earthquake of 2011.

[4] Lubben JE, Gironda ME (2003). Centrality of social ties to the health and well-being of older adults. Social work and health care in an aging world.

[5] Lubben JE (1988). Assessing social networks among elderly populations. Fam Commun Health.

[6] Steptoe A, Shankar A, Demakakos P, Wardle J (2013). Social isolation, loneliness, and all-cause mortality in older men and women. Proc Natl Acad Sci U S A.

[7] Kaniasty K, Norris FH (1993). A test of the social support deterioration model in the context of natural disaster. J Pers Soc Psychol.

[8] Shalev AY (2002). Acute stress reactions in adults. Biol Psychiatry.

[9] Yokoyama Y, Otsuka K, Kawakami N, Kobayashi S, Ogawa A, Tannno K, Onoda T, Yaegashi Y, Sakata K (2014). Mental health and related factors after the great East Japan earthquake and tsunami. PLoS One.

[10] Sone T, Nakaya N, Sugawara Y, Tomata Y, Watanabe T, Tsuji I (2016). Longitudinal association between time-varying social isolation and psychological distress after the great East Japan earthquake. Soc Sci Med.

[11] Brummett BH, Barefoot JC, Siegler IC, Clapp-Channing NE, Lytle BL, Bosworth HB, Williams RB, Mark DB (2001). Characteristics of socially isolated patients with coronary artery disease who are at elevated risk for mortality. Psychosom Med.

[12] Barth J, Schneider S, von Känel R (2010). Lack of social support in the etiology and the prognosis of coronary heart disease: a systematic review and meta-analysis. Psychosom Med.

[13] Cohen S, Doyle WJ, Skoner DP, Rabin BS, Gwaltney JM (1997). Social ties and susceptibility to the common cold. JAMA.

[14] Steptoe A, Owen N, Kunz-Ebrecht SR, Brydon L (2004). Loneliness and neuroendocrine, cardiovascular, and inflammatory stress responses in middle-aged men and women. Psychoneuroendocrinology.

[15] Bassuk SS, Glass TA, Berkman LF (1999). Social disengagement and incident cognitive decline in community-dwelling elderly persons. Ann Intern Med.

[16] Wilson RS, Krueger KR, Arnold SE, Schneider JA, Kelly JF, Barnes LL, Tang Y, Bennett DA (2007). Loneliness and risk of Alzheimer disease. Arch Gen Psychiatry.

[17] Cacioppo JT, Hughes ME, Waite LJ, Hawkley LC, Thisted RA (2006). Loneliness as a specific risk factor for depressive symptoms: cross-sectional and longitudinal analyses. Psychol Aging.

[18] Cacioppo JT, Hawkley LC, Thisted RA (2010). Perceived social isolation makes me sad: 5-year cross-lagged analyses of loneliness and depressive symptomatology in the Chicago health, aging, and social relations study. Psychol Aging.

[19] American Psychiatric Association (2013). Diagnostic and statistical manual of mental disorders (DSM-5).

[20] Kessler RC, Berglund P, Demler O, Jin R, Merikangas KR, Walters EE (2005). Lifetime prevalence and age-of-onset distributions of DSM-IV disorders in the national comorbidity survey replication. Arch Gen Psychiatry.

[21] Weissman MM, Bland RC, Canino GJ, Faravelli C, Greenwald S, Hwu HG, Joyce PR, Karam EG, Lee CK, Lellouch J, Lépine JP, Newman SC, Rubio-Stipec M, Wells JE, Wickramaratne PJ, Wittchen H, Yeh EK (1996). Cross-national epidemiology of major depression and bipolar disorder. JAMA.

[22] Guo S, Tian D, Wang X, Xiao Y, He H, Qu Z, Zhang X (2015). Protective effects of social support content and support source on depression and its prevalence 6 months after Wenchuan earthquake. Stress Health.

[23] Matsubara C, Murakami H, Imai K, Mizoue T, Akashi H, Miyoshi C, Nakasa T (2014). Prevalence and risk factors for depressive reaction among resident survivors after the tsunami following the great East Japan earthquake, march 11, 2011. PLoS One.

[24] Goenjian AK, Najarian LM, Pynoos RS, Steinberg AM, Manoukian G, Tavosian A, Fairbanks LA (1994). Posttraumatic stress disorder in elderly and younger adults after the 1988 earthquake in Armenia. Am J Psychiatr.

[25] Tsuboya T, Aida J, Hikichi H, Subramanian SV, Kondo K, Osaka K, Kawachi I (2016). Predictors of depressive symptoms following the great East Japan earthquake: a prospective study. Soc Sci Med.

[26] Cao H, McFarlane AC, Klimidis S (2003). Prevalence of psychiatric disorder following the 1988 Yun Nan (China) earthquake--the first 5-month period. Soc Psychiatry Psychiatr Epidemiol.

[27] Kuriyama S, Yaegashi N, Nagami F, Arai T, Kawaguchi Y, Osumi N, Sakaida M, Suzuki Y, Nakayama K, Hashizume H, Tamiya G, Kawame H, Suzuki K, Hozawa A, Nakaya N, Kikuya M, Metoki H, Tsuji I, Fuse N, Kiyomoto H, Sugawara J, Tsuboi A, Egawa S, Ito K, Chida K, Ishii T, Tomita H, Taki Y, Minegishi N, Ishii N, Yasuda J, Igarashi K, Shimizu R, Nagasaki M, Koshiba S, Kinoshita K, Ogishima S, Takai-Igarashi T, Tominaga T, Tanabe O, Ohuchi N, Shimosegawa T, Kure S, Tanaka H, Ito S, Hitomi J, Tanno K, Nakamura M, Ogasawara K, Kobayashi S, Sakata K, Satoh M, Shimizu A, Sasaki M, Endo R, Sobue K, Tohoku Medical Megabank Project Study Group, Yamamoto M (2016). The Tohoku medical megabank project: design and mission. J Epidemiol.

[28] Hozawa A, Tanno K, Nakaya N, Nakamura T, Tsuchiya N, Hirata T, Narita A, Kogure M, Nochioka K, Sasaki R, Takanashi N, Otsuka K, Sakata K, Kuriyama S, Kikuya M, Tanabe O, Sugawara J, Suzuki K, Suzuki Y, Kodama EN, Fuse N, Kiyomoto H, Tomita H, Uruno A, Hamanaka Y, Metoki H, Ishikuro M, Obara T, Kobayashi T, Kitatani K, Takai-Igarashi T, Ogishima S, Satoh M, Ohmomo H, Tsuboi A, Egawa S, Ishii T, Ito K, Ito S, Taki Y, Minegishi N, Ishii N, Nagasaki M, Igarashi K, Koshiba S, Shimizu R, Tamiya G, Nakayama K, Motohashi H, Yasuda J, Shimizu A, Hachiya T, Shiwa Y, Tominaga T, Tanaka H, Oyama K, Tanaka R, Kawame H, Fukushima A, Ishigaki Y, Tokutomi T, Osumi N, Kobayashi T, Nagami F, Hashizume H, Arai T, Kawaguchi Y, Higuchi S, Sakaida M, Endo R, Nishizuka S, Tsuji I, Hitomi J, Nakamura M, Ogasawara K, Yaegashi N, Kinoshita K, Kure S, Sakai A, Kobayashi S, Sobue K, Sasaki M, Yamamoto M (2021). Study profile of the Tohoku medical megabank community-based cohort study. J Epidemiol.

[29] Radloff LS (1977). The CES-D scale: a self-report depression scale for research in the general population. Appl Psychol Meas.

[30] Shima S, Shikano T, Kitamura T, Asai M (1985). A new self-report depression scale. Seishin Igaku.

[31] Lewinsohn PM, Seeley JR, Roberts RE, Allen NB (1997). Center for Epidemiologic Studies Depression Scale (CES-D) as a screening instrument for depression among community-residing older adults. Psychology and aging. Center Epidemiol Stud Depress Scale.

[32] Lubben J, Blozik E, Gillmann G, Iliffe S, von Renteln KW, Beck JC, Stuck AE (2006). Performance of an abbreviated version of the Lubben social network scale among three European Community-dwelling older adult populations. The Gerontologist.

[33] Kurimoto A, Awata S, Ohkubo T, Tsubota-Utsugi M, Asayama K, Takahashi K, Suenaga K, Satoh H, Imai Y (2011). Reliability and validity of the Japanese version of the abbreviated Lubben social network scale. Nihon Ronen Igakkai zasshi. Nihon Ronen Igakkai Zasshi. Jpn J Geriatr.

[34] Pearlin LI, Johnson JS (1977). Marital status, life-strains and depression. Am Sociol Rev.

[35] Glassman AH, Helzer JE, Covey LS, Cottler LB, Stetner F, Tipp JE, Johnson J (1990). Smoking, smoking cessation, and major depression. JAMA.

[36] Greenfield SF, Weiss RD, Muenz LR, Vagge LM, Kelly JF, Bello LR, Michael J (1998). The effect of depression on return to drinking: a prospective study. Arch Gen Psychiatry.

[37] Taylor DJ, Lichstein KL, Durrence HH, Reidel BW, Bush AJ (2005). Epidemiology of insomnia, depression, and anxiety. Sleep.

[38] Soldatos CR, Dikeos DG, Paparrigopoulos TJ (2000). Athens insomnia scale: validation of an instrument based on ICD-10 criteria. J Psychosom Res.

[39] Okajima I, Nakajima S, Kobayashi M, Inoue Y (2013). Development and validation of the Japanese version of the Athens insomnia scale. Psychiatry Clin Neurosci.

[40] Ginexi EM, Weihs K, Simmens SJ, Hoyt DR (2000). Natural disaster and depression: a prospective investigation of reactions to the 1993 Midwest floods. Am J Community Psychol.

[41] Cho S, Cho Y (2017). Depressive symptoms following natural disaster in Korea: psychometric properties of the Center for Epidemiologic Studies Depression Scale. Health Qual Life Outcomes.

[42] Sasaki Y, Aida J, Tsuji T, Koyama S, Tsuboya T, Saito T (2019). Pre-disaster social support is protective for onset of post-disaster depression: Prospective study from the Great East Japan Earthquake & Tsunami. 19427. Sci Rep.

[43] Bland SH, O’Leary ES, Farinaro E, Jossa F, Krogh V, Violanti JM, Trevisan M (1997). Socialnetwork disturbances and psychological distress following earthquake evacuation. J Nerv Ment Dis.

[44] Seplaki CL, Goldman N, Weinstein M, Lin YH (2006). Before and after the 1999 chi-chi earthquake: traumatic events and depressive symptoms in an older population. Soc Sci Med.

[45] Seto M, Nemoto H, Kobayashi N, Kikuchi S, Honda N, Kim Y, Kelman I, Tomita H (2019). Post-disastermental health and psychosocial support in the areas affected by the great East Japan earthquake: a qualitative study. BMC Psychiatry.

[46] Nakaya N, Nakamura T, Tsuchiya N, Narita A, Tsuji I, Hozawa A, Tomita H (2016). Housing prospect and distress. Psychiatry Clin Neurosci.

