# Supplementary material for: Association between the social isolation and depressive symptoms after the great East Japan earthquake: findings from the baseline survey of the TMM CommCohort study
Source: BMC Public Health. 2021 May 15;21:925. doi: 10.1186/s12889-021-10896-5 (PMC8122535; doi:10.1186/s12889-021-10896-5)
Supplement: Supplementary file 1 — Additional file 1: Supplemental Table 1. Comparison of sociodemographics between participants and nonparticipants. Supplemental Table 2. Proportion of depressive symptoms by survey year and area between Iwate and Miyagi prefectures. Supplemental Table 3. Proportion of depressive symptoms monthly and by season. Supplemental Table 4. Adjusted ORs (95% CI) of depressive symptoms according to social isolation by survey year. Supplemental Table 5. Adjusted ORs (95% CI) of depressive symptoms according to social isolation by age group. Supplemental Table 6. Adjusted ORs (95% CI) of depressive symptoms according to house damage, death of family members, and social isolation by sex by analyzing multiply imputed datasets. Supplemental Table 7. Adjusted ORs (95% CI) of depressive symptoms according to social isolation by survey year by analyzing multiply imputed datasets. Supplemental Table 8. Adjusted ORs (95% CI) of depressive symptoms according to social isolation by age group by analyzing multiply imputed datasets. Supplemental Table 9. Adjusted ORs (95% CI) of depressive symptoms according to the severity of house damage and social isolation by analyzing multiply imputed datasets. Supplemental Table 10. Adjusted ORs (95% CI) of depressive symptoms according to death of family members due to the GEJE and social isolation by analyzing multiply imputed datasets. [file 12889_2021_10896_MOESM1_ESM.docx]

**Association between the social isolation and depressive symptoms after the Great East Japan Earthquake: Findings from the baseline survey of the TMM CommCohort Study**

Yuka Kotozaki^1^, Kozo Tanno^1,2^, Kiyomi Sakata^1,2^, Eri Takusari^2^, Kotaro Otsuka^1,3^, Hiroaki Tomita^4,5,6^, Ryohei Sasaki^7^, Nobuyuki Takanashi^1,2^, Takahiro Mikami^1,8^, Atsushi Hozawa^5^, Naoki Nakaya^5,9^, Naho Tsuchiya^5^, Tomohiro Nakamura^5^, Akira Narita^5^, Yasuyuki Taki^5,10^, Atsushi Shimizu^1,11^, Jiro Hitomi^8^, Mamoru Satoh^1,11^, Makoto Sasaki^1,12^.

Institutions:

*1. Iwate Tohoku Medical Megabank Organization, Iwate Medical University, Iwate, Japan*

*2. Department of Hygiene and Preventive Medicine, School of Medicine, Iwate Medical University, Iwate, Japan*

*3. Department of Neuropsychiatry, School of Medicine, Iwate Medical University, Iwate, Japan*

*4. Department of Psychiatry, Graduate School of Medicine, Tohoku University, Sendai, Japan*

*5. Tohoku Medical Megabank Organization, Tohoku University, Sendai, Japan*

*6. Division of Disaster Medical Science, International Research Institute of Disaster Science, Tohoku University, Sendai, Japan*

*7. Division of Physical Education, Department of Human Sciences, Iwate Medical University Center for Liberal Arts and Sciences, Iwate, Japan*

*8. Department of Anatomy, School of Medicine, Iwate Medical University, Iwate, Japan*

*9. Department of Health Science, Saitama Prefectural University, Koshigaya, Japan*

*10. Department of Radiology and Nuclear Medicine, Institute of Development, Aging and Cancer, Tohoku University, Sendai, Japan*

*11. Division of Biomedical Information Analysis, Institute for Biomedical Sciences, Iwate Medical University, Iwate, Japan*

*12. Division of Ultrahigh Field MRI, Institute for Biomedical Sciences, Iwate Medical University, Iwate, Japan*

Corresponding author: Yuka Kotozaki, Ph.D.

Address: Division of Clinical Research and Epidemiology, Iwate Tohoku Medical Megabank Organization, Iwate Medical University.

1-1-1 Idaidori, Yahaba, Shiwa, Iwate 028-3694, Japan

Telephone numbers: +81 19 651 5110

E-mail: kotoyuka@iwate-med.ac.jp

Key words:

Tohoku Medical Megabank Project, Cross-sectional study, Great East Japan Earthquake, House damage, Death of family members, Social isolation, Depressive symptoms

Short running title: Social isolation and depressive symptoms: Baseline survey of TMM CommCohort Study

Numbers of Tables 4, Figures 1, Supplementary materials 10.

**Supplemental Table 1.** Comparison of sociodemographics between participants and nonparticipants.

|  |  | Participants  (n = 48,958) | Nonparticipants  (n = 14,225) | *P* Value | Missing  Value |
| --- | --- | --- | --- | --- | --- |
| Age (continuous) |  | 60.5 (11.2) | 60.6 (11.2) | 0.790* | 0 |
| Gender (females, %) |  | 61.7 | 61.7 | 0.983 | 0 |
| Survey year (%) | 2013 | 25.1 | 24.8 | 0.853 | 0 |
|  | 2014 | 40.9 | 40.7 |  |  |
|  | 2015 | 34.1 | 34.5 |  |  |
| Area (%) | Inland | 47.7 | 47.8 | 0.865 | 0 |
|  | Coast | 52.3 | 52.2 |  |  |
| Depressive symptoms (%) |  | 26.5 | 26.1 | 0.391 | 7401 |
| Social isolation (%) |  | 26.3 | 25.8 | 0.285 | 6627 |
| Severity of house damage (%) | Undamaged | 46.3 | 47.0 | 0.357** | 6472 |
|  | Half-damaged | 43.7 | 43.3 |  |  |
|  | Totally damaged | 10.0 | 9.7 |  |  |
| Death of family members due to the GEJE (%) | One or more | 38.5 | 38.8 | 0.559 | 6998 |
| Education level (%) | Junior high school | 22.8 | 23.4 | 0.173 | 4136 |
|  | High school | 68.7 | 68.4 |  |  |
|  | College, university, and higher | 7.7 | 7.4 |  |  |
|  | Other | 0.8 | 0.9 |  |  |
| Marital status (%) | Unmarried | 20.6 | 21.3 | 0.081 | 4617 |
| Number of household members (%) | Living alone | 7.1 | 7.0 | 0.571 | 5034 |
| Working status (%) | Unemployed | 51.0 | 50.9 | 0.895 | 4836 |
| Smoking habits (%) | Smoker | 14.1 | 14.4 | 0.379 | 4627 |
| Drinking habits (%) | Drinker | 49.3 | 48.8 | 0.382 | 4028 |
| Past or current major illness (%) | Hypertension | 28.0 | 29.0 | 0.009 | 0 |
|  | Diabetes mellitus | 6.8 | 7.0 | 0.272 | 0 |
|  | Hyperlipidemia | 14.0 | 13.4 | 0.097 | 0 |
|  | Cancer | 6.9 | 7.2 | 0.333 | 0 |
|  | Coronary artery disease | 2.4 | 2.2 | 0.136 | 0 |
|  | Stroke | 2.0 | 2.0 | 0.736 | 0 |
| BMI (%) | <18.5 kg/m^2^ | 5.3 | 5.2 | 0.308 | 1067 |
|  | 18.5 to <25.0 kg/m^2^ | 64.4 | 63.8 |  |  |
|  | ≥25.0 kg/m^2^ | 30.3 | 31.0 |  |  |
| Insomnia (%) |  | 23.0 | 22.8 | 0.626 | 4704 |

Depressive symptoms, CES-D ≥ 16; social isolation, LSNS-6 < 12; insomnia, AIS ≥ 6.

CES-D, Center for Epidemiologic Studies Depression Scale; LSNS-6, Lubben Social Network Scale-6; AIS, Athene Insomnia Scale; GEJE, Great East Japan Earthquake; BMI, body mass index.

*P* value: tested by chi-square test; * tested by student t-test; ** tested by chi-square test for linear trend.

Statistical significance, *P* < 0.05.

**Supplemental Table 2.** Proportion of depressive symptoms by survey year and area between Iwate and Miyagi prefectures.

|  | Iwate | | | Miyagi | | |
| --- | --- | --- | --- | --- | --- | --- |
|  | Inland | Coast | *P* Value | Inland | Coast | *P* Value |
| 2013 | 26.9 | 25.4 | 0.427 | 23.3 | 29.3 | <0.001 |
| 2014 | 24.6 | 27.7 | 0.107 | 23.9 | 25.1 | 0.098 |
| 2015 | 22.6 | 27.2 | <0.001 | 24.0 | 26.4 | 0.042 |

**Supplemental Table 3.** Proportion of depressive symptoms monthly and by season.

a) Monthly

|  | Monthly | | | | | | | | | |
| --- | --- | --- | --- | --- | --- | --- | --- | --- | --- | --- |
|  | April | May | June | July | August | September | October | November | December | January |
| Depressive symptoms (%) | 24.2 | 26.3 | 23.6 | 26.1 | 26.2 | 24.9 | 24.4 | 25.8 | 26.3 | 20.5 |

b) By the season

|  | Spring  (April to May) | Summer  (June to August) | Autumn  (September to November) | Winter  (December to January) | *P* Value |
| --- | --- | --- | --- | --- | --- |
| Depressive symptoms (%) | 25.3 | 25.3 | 25.0 | 23.4 | 0.740 |

**Supplemental Table 4.** Adjusted ORs (95% CI) of depressive symptoms according to social isolation by survey year.

|  |  | Males (n = 18,423) | | | |  |  | Females (n = 30,535) | | | |
| --- | --- | --- | --- | --- | --- | --- | --- | --- | --- | --- | --- |
|  |  | OR | 95% CI | *P* Value | *P* for interaction |  |  | OR | 95% CI | *P* Value | *P* for Interaction |
| 2013  (n = 4,251) |  |  | | | 0.563 | 2013  (n = 7,214) |  |  | | | 0.444 |
|  | No. of Cases with Depressive Symptoms / No. of Subjects |  |  |  |  |  | No. of Cases with Depressive Symptoms / No. of Subjects |  |  |  |  |
| Social isolation | 374 / 1143 | 2.06 | 1.72 – 2.47 | <0.001 |  | Social isolation | 716 / 1617 | 2.14 | 1.88 – 2.44 | <0.001 |  |
| Nonsocial isolation | 498 / 3108 | 1.00 | reference |  |  | Nonsocial isolation | 1421 / 5597 | 1.00 | reference |  |  |
|  |  |  |  |  |  |  |  |  |  |  |  |
| 2014  (n = 7690) |  |  |  |  |  | 2014  (n = 12,729) |  |  |  |  |  |
|  | No. of cases with Depressive Symptoms / No. of Subjects |  |  |  |  |  | No. of Cases with Depressive Symptoms / No. of Subjects |  |  |  |  |
| Social isolation | 669 / 2,256 | 1.87 | 1.63 – 2.13 | <0.001 |  | Social isolation | 1350 / 3105 | 2.06 | 1.87 – 2.26 | <0.001 |  |
| Nonsocial isolation | 865 / 5434 | 1.00 | reference |  |  | Nonsocial isolation | 2304 / 9624 | 1.00 | reference |  |  |
|  |  |  |  |  |  |  |  |  |  |  |  |
| 2015  (n = 6,482) |  |  |  |  |  | 2015  (n = 10,592) |  |  |  |  |  |
|  | No. of cases with Depressive Symptoms / No. of Subjects |  |  |  |  |  | No, of Cases with Depressive Symptoms / No. of Subjects |  |  |  |  |
| Social isolation | 549 / 1,910 | 1.82 | 1.57 - 2.11 | <0.001 |  | Social isolation | 1,111 / 2,573 | 2.22 | 2.00 - 2.47 | <0.001 |  |
| Nonsocial isolation | 691 / 4,572 | 1.00 | reference |  |  | Nonsocial isolation | 1,849 / 8,019 | 1.00 | reference |  |  |

OR, odds ratio; 95% CI, 95% confidence interval.

Depressive symptoms, CES-D ≥ 16; social isolation, LSNS-6 <12.

Adjusted for age; survey year; area; education level; marital status; number of household members; working status; smoking habits; drinking habits; past or current major illness; BMI; AIS; severity of house damage; death of family members due to the GEJE; and survey year*area.

Statistical significance, *P* < 0.05.

**Supplemental Table 5.** Adjusted ORs (95% CI) of depressive symptoms according to social isolation by age group.

|  |  | Males (n = 18,423) | | | |  |  | Females (n = 30,535) | | | |
| --- | --- | --- | --- | --- | --- | --- | --- | --- | --- | --- | --- |
|  |  | OR | 95% CI | *P* Value | *P* for Interaction |  |  | OR | 95% CI | *P* Value | *P* for Interaction |
| age <65  (n = 8,512) |  |  | | | 0.005 | age <65  (n = 18,830) |  |  |  |  | 0.635 |
|  | No. of Cases with Depressive Symptoms / No. of Subjects |  |  |  |  |  | No. of Cases with Depressive Symptoms / No. of Subjects |  |  |  |  |
| Social isolation | 977 / 2878 | 2.10 | 1.86 – 2.37 | <0.001 |  | Social isolation | 2354 / 5161 | 2.11 | 1.95 – 2.27 | <0.001 |  |
| Nonsocial isolation | 924 / 5634 | 1.00 | reference |  |  | Nonsocial isolation | 3549 / 13,669 | 1.00 | reference |  |  |
|  |  |  |  |  |  |  |  |  |  |  |  |
| age ≥65  (n = 9911) |  |  |  |  |  | age ≥65  (n = 11,705) |  |  |  |  |  |
|  | No. of Cases with Depressive Symptoms / No. of Subjects |  |  |  |  |  | No. of Cases with Depressive Symptoms / No. of Subjects |  |  |  |  |
| Social isolation | 615 / 2431 | 1.68 | 1.49 – 1.91 | <0.001 |  | Social isolation | 830 / 2134 | 2.15 | 1.92 – 2.40 | <0.001 |  |
| Nonsocial isolation | 1130 / 7480 | 1.00 | reference |  |  | Nonsocial isolation | 2025 / 9571 | 1.00 | reference |  |  |

Depressive symptoms, CES-D ≥ 16; social isolation, LSNS-6 <12.

Adjusted for age; survey year; area; education level; marital status; number of household members; working status; smoking habits; drinking habits; past or current major illness; BMI; AIS; severity of house damage; death of family members due to the GEJE; and survey year*area.

Statistical significance, *P* < 0.05.

**Supplemental Table 6.** Adjusted ORs (95% CI) of depressive symptoms according to house damage, death of family members, and social isolation by sex by analyzing multiply imputed datasets.

|  | Male (n = 24,208) | | | |  | Female (n = 39,005) | | | |
| --- | --- | --- | --- | --- | --- | --- | --- | --- | --- |
|  | No. of Cases with Depressive Symptoms / No. of Subjects | OR | 95% CI | *P* Value |  | No. of Cases with Depressive Symptoms / No. of Subjects | OR | 95% CI | *P* Value |
| House damage |  |  |  |  |  |  |  |  |  |
| Undamaged | 2258 / 11,142 | 1.00 | reference |  |  | 4997 / 18,329 | 1.00 | reference |  |
| Half-damaged | 2301 / 10,736 | 1.16 | 1.13 – 1.20 | <0.001 |  | 4953 / 16,714 | 1.15 | 1.12 – 1.18 | <0.001 |
| Totally damaged | 737 / 2330 | 1.61 | 1.53 – 1.70 | <0.001 |  | 1549 / 3962 | 1.43 | 1.38 – 1.49 | <0.001 |
| Death of family members | 2256 / 9508 | 1.15 | 1.11 – 1.19 | <0.001 |  | 4846 / 14,848 | 1.24 | 1.21 –1.27 | <0.001 |
| Social isolation | 2306 / 6954 | 1.90 | 1.84 – 1.96 | <0.001 |  | 4217 / 9450 | 2.14 | 2.09 – 2.20 | <0.001 |

OR, odds ratio; 95% CI, 95% confidence interval.

Depressive symptoms, CES-D ≥ 16; social isolation, LSNS-6 <12.

Adjusted for age; survey year; area; education level; marital status; number of household members; working status; smoking habits; drinking habits; past or current major illness; BMI; AIS; and survey year*area.

Statistical significance, *P* < 0.05.

**Supplemental Table 7.** Adjusted ORs (95% CI) of depressive symptoms according to social isolation by survey year by analyzing multiply imputed datasets.

|  |  | Males (n = 24,208) | | | |  |  | Females (n = 39,005) | | | |
| --- | --- | --- | --- | --- | --- | --- | --- | --- | --- | --- | --- |
|  |  | OR | 95% CI | *P* Value | *P* for Interaction |  |  | OR | 95% CI | *P* Value | *P* for Interaction |
| 2013  (n = 5,822) |  |  | | | 0.564 | 2013  (n = 9839) |  |  | | | 0.444 |
|  | No. of Cases with Depressive Symptoms / No. of Subjects |  |  |  |  |  | No. of Cases with Depressive Symptoms / No. of Subjects |  |  |  |  |
| Social isolation | 562 / 1597 | 2.00 | 1.87 – 2.14 | <0.001 |  | Social isolation | 1621 / 2268 | 2.18 | 2.07 – 2.29 | <0.001 |  |
| Nonsocial isolation | 741 / 4225 | 1.00 | reference |  |  | Nonsocial isolation | 1943 / 7571 | 1.00 | reference |  |  |
|  |  |  |  |  |  |  |  |  |  |  |  |
| 2014  (n = 9978) |  |  |  |  |  | 2014  (n = 15,977) |  |  |  |  |  |
|  | No. of Cases with Depressive Symptoms / No. of Subjects |  |  |  |  |  | No. of Cases with Depressive Symptoms / No. of Subjects |  |  |  |  |
| Social isolation | 967 / 2891 | 1.93 | 1.54 – 2.04 | <0.001 |  | Social isolation | 1741 / 3909 | 2.10 | 2.02 – 2.18 | <0.001 |  |
| Nonsocial isolation | 1238 / 7087 | 1.00 | reference |  |  | Nonsocial isolation | 2921 / 12,068 | 1.00 | reference |  |  |
|  |  |  |  |  |  |  |  |  |  |  |  |
| 2015  (n = 8408) |  |  |  |  |  | 2015  (n = 13,189) |  |  |  |  |  |
|  | No. of Cases with Depressive Symptoms / No. of Subjects |  |  |  |  |  | No. of Cases with Depressive Symptoms / No. of Subjects |  |  |  |  |
| Social isolation | 777 / 5942 | 1.85 | 1.75 – 1.95 | <0.001 |  | Social isolation | 1455 / 3273 | 2.19 | 2.10 – 2.29 | <0.001 |  |
| Nonsocial isolation | 1011 / 5942 | 1.00 | reference |  |  | Nonsocial isolation | 2418 / 9916 | 1.00 | reference |  |  |

OR, odds ratio; 95% CI, 95% confidence interval.

Depressive symptoms, CES-D ≥ 16; social isolation, LSNS-6 <12.

Adjusted for age; survey year; area; education level; marital status; number of household members; working status; smoking habits; drinking habits; past or current major illness; BMI; AIS; severity of house damage; death of family members due to the GEJE; and survey year*area.

Statistical significance, *P* < 0.05.

**Supplemental Table 8.** Adjusted ORs (95% CI) of depressive symptoms according to social isolation by age group by analyzing multiply imputed datasets.

|  |  | Males (n = 24,208) | | | |  |  | Females (n = 39,005) | | | |
| --- | --- | --- | --- | --- | --- | --- | --- | --- | --- | --- | --- |
|  |  | OR | 95% CI | *P* Value | *P* for Interaction |  |  | OR | 95% CI | *P* Value | *P* for Interaction |
| age <65  (n = 11,029) |  |  | | | 0.005 | age <65  (n = 22,801) |  |  |  |  | 0.635 |
|  | No. of Cases with Depressive Symptoms / No. of Subjects |  |  |  |  |  | No. of Cases with Depressive Symptoms / No. of Subjects |  |  |  |  |
| Social isolation | 1338 / 3661 | 2.01 | 1.92 – 2.11 | <0.001 |  | Social isolated | 2946 / 6280 | 2.11 | 2.05 – 2.18 | <0.001 |  |
| Nonsocial isolation | 1372 / 7368 | 1.00 | reference |  |  | Nonsocial isolation | 4362 / 16,521 | 1.00 | reference |  |  |
|  |  |  |  |  |  |  |  |  |  |  |  |
| age ≥65  (n = 13,179) |  |  |  |  |  | age ≥65  (n = 16,204) |  |  |  |  |  |
|  | No. of Cases with Depressive Symptoms / No. of Subjects |  |  |  |  |  | No. of Cases with Depressive Symptoms / No. of Subjects |  |  |  |  |
| Social isolation | 968 /3293 | 1.80 | 1.72 – 1.89 | <0.001 |  | Social isolation | 1271 / 3180 | 2.20 | 2.11 – 2.24 | <0.001 |  |
| Nonsocial isolated | 1618 / ,886 | 1.00 | reference |  |  | Nonsocial isolation | 2920 / 13,034 | 1.00 | reference |  |  |

Depressive symptoms, CES-D ≥ 16; social isolation, LSNS-6 <12.

Adjusted for age; survey year; area; education level; marital status; number of household members; working status; smoking habits; drinking habits; past or current major illness; BMI; AIS; severity of house damage; death of family members due to the GEJE; and survey year*area.

Statistical significance, *P* < 0.05.

**Supplemental Table 9.** Adjusted ORs (95% CI) of depressive symptoms according to the severity of house damage and social isolation by analyzing multiply imputed datasets.

|  | Males (n = 24,208) | | | | | Females (n = 39,005) | | | | |
| --- | --- | --- | --- | --- | --- | --- | --- | --- | --- | --- |
| The severity of house damage╳social isolation | No. of Cases with Depressive Symptoms / No. of Subjects | OR | 95% CI | *P* Value | *P* for Interaction | No. of Cases with Depressive Symptoms / No. of Subjects | OR | 95% CI | *P* Value | *P* for Interaction |
| Undamaged ╳Nonsocial isolation | 1223 / 7759 | 1.00 | reference |  | 0.442 | 3007 / 13,505 | 1.00 | reference |  | 0.407 |
| Half-damaged ╳Nonsocial isolation | 1335 / 7831 | 1.15 | 1.10 – 1.20 | <0.001 |  | 3254 / 13,065 | 1.13 | 1.10 – 1.16 | <0.001 |  |
| Totally-damaged ╳Nonsocial isolation | 422 / 1664 | 1.55 | 1.49 – 1.66 | <0.001 |  | 1021 / 2985 | 1.46 | 1.40 – 1.53 | <0.001 |  |
| Undamaged ╳Social isolation | 1025 / 3383 | 1.85 | 1.77 – 1.94 | <0.001 |  | 1990 / 4824 | 2.11 | 2.04 – 2.19 | <0.001 |  |
| Half-damaged ╳Social isolation | 966 / 2905 | 2.20 | 2.09 – 2.31 | <0.001 |  | 1699 / 3649 | 2.52 | 2.43 – 2.62 | <0.001 |  |
| Totally damaged ╳Social isolation | 315 / 666 | 3.20 | 2.94 – 3.48 | <0.001 |  | 528 / 977 | 2.87 | 2.68 – 3.07 | <0.001 |  |

OR, odds ratio; 95% CI, 95% confidence interval.

Depressive symptoms, CES-D ≥ 16; social isolation, LSNS-6 <12.

Adjusted for age; survey year; area; education level; marital status; number of household members; working status; smoking habits; drinking habits; past or current major illness; BMI; AIS; death of family members due to the GEJE; and survey year*area.

Statistical significance, *P* < 0.05.

**Supplemental Table 10.** Adjusted ORs (95% CI) of depressive symptoms according to death of family members due to the GEJE and social isolation by analyzing multiply imputed datasets.

|  | Males (n = 24,208) | | | | | Females (n = 39,005) | | | | |
| --- | --- | --- | --- | --- | --- | --- | --- | --- | --- | --- |
| The death of family members due to the GEJE╳Social isolation | No of cases with depressive symptoms / No of subjects | OR | 95% CI | *P* Value | *P* for Interaction | No. of Cases with Depressive Symptoms / No. of Subjects | OR | 95% CI | *P* Value | *P* for Interaction |
| No death of family members due to the GEJE ╳Nonsocial isolation | 1597 / 10,072 | 1.00 | reference |  | 0.853 | 3993 / 17,806 | 1.00 | reference |  | 0.543 |
| Death of family members due to the GEJE ╳Nonsocial isolation | 1393 / 7162 | 1.14 | 1.09 – 1.18 | <0.001 |  | 3289 / 11,749 | 1.23 | 1.20 – 1.27 | <0.001 |  |
| No death of family members due to the GEJE  ╳Social isolation | 1443 / 4628 | 1.88 | 1.80 – 1.96 | <0.001 |  | 2660 / 6351 | 2.13 | 2.07 – 2.20 | <0.001 |  |
| Death of family members due to the GEJE ╳Social isolation | 863 / 2,326 | 2.20 | 2.09 - 2.32 | <0.001 |  | 1557 / 3,099 | 2.67 | 2.56 - 2.78 | <0.001 |  |

OR, odds ratio; 95% CI, 95% confidence interval.

Depressive symptoms, CES-D ≥ 16; social isolation, LSNS-6 <12.

Adjusted for age; survey year; area; education level; marital status; number of household members; working status; smoking habits; drinking habits; past or current major illness; BMI; AIS; severity of house damage; and survey year*area.

Statistical significance, *P* < 0.05.
